# Editorial: Neonatal sepsis: current insights and challenges

**DOI:** 10.3389/fped.2024.1427503

**Published:** 2024-05-29

**Authors:** Rozeta Sokou, Stavroula Parastatidou, Aikaterini Konstantinidi, Andreas G. Tsantes, Nicoletta Iacovidou

**Affiliations:** ^1^ Neonatal Intensive Care Unit, Nikaia General Hospital “Aghios Panteleimon”, Piraeus, Greece; ^2^ Department of Neonatology Aretaieio Hospital, National and Kapodistrian University of Athens, Athens, Greece; ^3^ Neonatal Intensive Care Unit, “Elena Venizelou” Maternity Hospital, Athens, Greece; ^4^ Microbiology Department, “Saint Savvas” Oncology Hospital, Athens, Greece

**Keywords:** neonatal sepsis, inflammatory biomarkers, neonates, bacterial species, necrotizing enterocolitis

**Editorial on the Research Topic** Neonatal sepsis: current insights and challenges

The incidence of bacterial sepsis ranges from 1 to 10 cases per 1,000 live births, with a 4-fold to 10-fold increase in preterm neonates (1–4). Despite the significant progress in neonatal care, sepsis remains a leading cause of morbidity and mortality in Neonatal Intensive Care Units (NICUs), accounting for 243,000 deaths per year globally (5–8).

The World Health Organization (WHO) recently identified the need to reduce the burden of neonatal sepsis as the Sustainable Development Goal 3, aiming to decrease neonatal mortality to at least 12 per 1,000 live births by 2030 (9). The reduction of sepsis-related deaths in low-and middle-income countries is essential in order to achieve this goal (10). The incidence of neonatal sepsis widely varies among different countries and territories, reflecting differences in health resources, maternal and neonatal risk factors, and prevention strategies (5, 11). National and regional data should be considered for the implementation of successful measures for decrease of the incidence and mortality of neonatal infections. Li et al., collected data from the Global Burden of Disease 2019 to evaluate global features of incidence and mortality of neonatal sepsis and other neonatal infections (NSNIs), and to guide global and regional interventions for prevention and control of NSNIs. From 1990 to 2019, NSNI cases presented an annual global increase of 12.79%, while NSNI deaths decreased by 12.93% annually. A demographic and health survey in Ethiopia (2016) reported a significant number of neonatal deaths related to sepsis (12). Ambaye et al. conducted a trial to evaluate the time to sepsis recovery and its defining factors among neonates admitted in Woldia Comprehensive Specialized Hospital (WCSH), Northeast Ethiopia. The results indicated that time to sepsis recovery is adversely and independently associated with induction of labor and resuscitation at birth. Gezmu et al. using data from a public, tertiary-level hospital in Botswana, evaluated multiple risk factors and identified sepsis as an independent risk factor for pulmonary hemorrhage. Candida infection ranks as the third most common cause of neonatal late-onset sepsis, with Candida albicans being the most frequently isolated species. However, there has been a recent increase in the incidence of non-albicans Candida sepsis (13), including Candida glabrata, a case of which is reported by Parramon-Teixido et al. describing a urinary tract infection in a preterm neonate.

Necrotizing enterocolitis (NEC) is another crucial complication that primarily affects premature infants and is characterized by inflammation, similar to sepsis. Additionally, both conditions present non-specific, often overlapping, clinical symptoms and signs, rendering their differential diagnosis difficult (14–16). The better comprehension of pathophysiological mechanisms of sepsis and NEC, essential for the improvement of timely and accurate diagnosis and treatment, is necessary. Jiang et al. evaluated the role of the proportion of large platelets (PLCR) and platelet crit (PCT) in prediction of NEC in low birth weight (LBW) neonates. Results of this study indicated that 2/100 LBW neonates were at risk of NEC and that sepsis and anemia were main factors associated with NEC. Stratification of confounding factors revealed the superiority of PLT activation (especially PLCR), compared to PLT count, in predicting NEC occurrence in non-septic, LBW neonates.

Early detection of neonatal sepsis is challenging (5). Delayed treatment increases mortality, while treating neonates with mild symptoms and signs or solely risk factors results in overtreatment and unnecessary use of antibiotics. The identification of an optimal biomarker for diagnosis and monitoring of neonatal sepsis is the target of ongoing research. Inflammatory biomarkers seem promising; yet, only a few have been incorporated in clinical practice. Kumar et al. analyzed hematologic and physiologic biomarkers of late-onset sepsis and NEC in very low birthweight neonates. A validated sepsis risk score (Pulse Oximetry Warning Score, POWS) (17) was used to test whether plasma biomarkers correlate with physiological biomarkers of sepsis and concluded that inflammatory biomarkers discriminated between late-onset septicemia due to Gram-negative or NEC and all other septic or non-septic conditions.

In general, the diagnostic tools currently used for sepsis are invasive and time-consuming. Newer, non-invasive analytical methods could detect an infection early and also identify the pathogen. Bous et al. developed a method to analyze the profile of volatile organic compounds of bacterial species. Multicapillary column-coupled ion mobility spectrometry was used and found appropriate for the identification and differentiation between specific bacteria, emerging as a useful *in vitro* diagnostic tool.

Purulent ophthalmic discharge poses a clinical dilemma in NICU setting, considering its potential association with late-onset sepsis. Gad et al. did not report a statistically significant correlation between purulent conjunctivitis with a positive swab culture and late-onset sepsis. Post-catheter removal sepsis (PCRS) is a notable complication of indwelling central venous catheters (CVCs) in neonates, attributed to disruption of biofilms formed along the catheter tip during removal of CVCs. The prevention of PCRS with the use of antibiotics during removal of CVCs remains debatable. Ji et al. conducted a meta-analysis, and according to their results, the use of antibiotics within 12 h of CVC removal does not significantly reduce PCRS, but is associated with reduced post-catheter removal blood stream infection.

Human milk has proved protective against neonatal sepsis and other infections, particularly in preterm neonates (18). A study by Moliner-Calderón et al. showed that any feeding with human milk was associated with a reduction in the need for vasoactive drugs in septic neonates, encouraging further research to clarify whether feeding with human milk directly affects neonatal patterns of cardiovascular maturation.

The present research topic has inspired significant focus on neonatal sepsis. Better understanding of this condition, its heterogeneity, epidemiology, and pathophysiology will help optimize short-term and long-term outcome and reduce burden on society. Neonatal sepsis has an impact that extends throughout life, and future studies should incorporate morbidity, mortality, long-term results and direct and indirect cost. This collection of sepsis-related articles in the current issue of “Frontiers of pediatrics” highlights the contemporary understanding and gaps and limitations in the diagnosis and treatment of sepsis, along with areas for further research. More studies are required on this topic, nonetheless, recent developments are quite promising and intriguing.
